# Antiretroviral Therapy for HIV-2 Infection: Recommendations for Management in Low-Resource Settings

**DOI:** 10.1155/2011/463704

**Published:** 2011-02-09

**Authors:** Kevin Peterson, Sabelle Jallow, Sarah L. Rowland-Jones, Thushan I. de Silva

**Affiliations:** ^1^Medical Research Council (UK) Laboratories, Atlantic Road, P.O. Box 273, Fajara, Gambia; ^2^Medical Research Council Human Immunology Unit, Weatherall Institute of Molecular Medicine, John Radcliffe Hospital, Oxford, UK; ^3^MRC/UCL Centre for Medical Molecular Virology, Division of Infection and Immunity, University College London, UK

## Abstract

HIV-2 contributes approximately a third to the prevalence of HIV in West Africa and is present in significant amounts in several low-income countries outside of West Africa with historical ties to Portugal. It complicates HIV diagnosis, requiring more expensive and technically demanding testing algorithms. Natural polymorphisms and patterns in the development of resistance to antiretrovirals are reviewed, along with their implications for antiretroviral therapy. Nonnucleoside reverse transcriptase inhibitors, crucial in standard first-line regimens for HIV-1 in many low-income settings, have no effect on HIV-2. Nucleoside analogues alone are not sufficiently potent enough to achieve durable virologic control. Some protease inhibitors, in particular those without ritonavir boosting, are not sufficiently effective against HIV-2. Following review of the available evidence and taking the structure and challenges of antiretroviral care in West Africa into consideration, the authors make recommendations and highlight the needs of special populations.

## 1. Introduction

HIV-2 represents a distinct lineage of HIV, stemming from SIVsm instead of the SIVcpz responsible for HIV-1. Like HIV-1 it appears to have made the transition to humans more than once, giving rise to eight distinct groups, of which groups A and B account for nearly all of the cases identified thus far [1]. HIV-2 differs from HIV-1 most strikingly in its lower rate of progression and infectivity, with the majority of those infected likely to be long-term nonprogressors [2–4]. Those with progressive disease experience the same likelihood of morbidity and mortality as are seen with HIV-1 [5, 6]. People with advanced HIV-2 infection require treatment with antiretroviral therapy (ART), but most individual antiretroviral drugs and regimens have been designed and optimized for HIV-1 and cannot be assumed to provide optimal viral suppression for HIV-2 infection. In some instances, antiretroviral susceptibility differs significantly between HIV-1 and HIV-2, such that HIV-2 is intrinsically resistant to two of the major classes of antiretroviral drugs: the fusion inhibitors and the nonnucleoside reverse transcriptase inhibitor- (NNRTI-) based regimens that are the standard therapy for HIV-1 in West Africa [7, 8]. 

The challenge of treating HIV-2 infection falls mainly upon West Africa [6], with current prevalence estimates ranging up to 1% where reported, compared with HIV-1 prevalence rates of up to 3.4%, therefore comprising a substantial portion of all HIV infections in the subregion [9]. The exception to this is Guinea-Bissau, where the prevalence amongst adults was estimated to be 8%–10% two decades ago [10]. This has now changed to a current prevalence of around 4%, compared to an HIV-1 prevalence of 2.9% in rural areas and 4.2% in urban areas [11–13]. European countries with colonial links to West Africa such as Portugal, France, and the United Kingdom, as well as other countries with prior Portuguese ties, such as Angola, Brazil, India, and Mozambique, also have sizeable cohorts of HIV-2 infected individuals [14–18]. Although the absolute numbers of patients infected with HIV-2 in European cohorts are small, the earlier availability of ART in these countries has provided some data to guide treatment recommendations in resource-poor settings. 

Given the prevalence of HIV-2 in West Africa, it is imperative that up-to-date recommendations be available for the antiretroviral management of HIV-2 in these clinical settings, characterized by the use of standardized first-, and in some cases second-line regimens based on limited formularies, with treatment decisions driven by protocol, that are also highly sensitive to cost. At the time of writing, therapeutic drug monitoring, viral load measurement, and genotypic resistance testing are not routinely available in West Africa, nor are coreceptor tropism assays or HLA typing (to guide the safe use of CCR5 receptor blockers or abacavir, resp.). The monitoring and care of HIV in sub-Saharan Africa has, however, been a litany of barriers brought down, and the “impossible” becomes the standard, so these recommendations seek to strike a balance between optimal and current management trends.

Clinical trials of ART in HIV-2 are few compared with HIV-1, primarily because of HIV-2's lower prevalence and virulence, not to mention its concentration among some of the world's poorest people. Until there is better evidence from randomized controlled trials, judgment of what constitutes good care in HIV-2 management must therefore rely on both *in vitro* as well as *in vivo* data from small cohort studies and case series, theoretical assertions, and parallels with HIV-1 therapeutics.

As will be apparent to experienced clinicians and program officers, numerous potential factors have been left out of this work that might influence program-level decisions about ART for HIV-2 in West Africa. This is especially true where such factors affect both HIV-1 and HIV-2 infections in the same way. The current work is not intended as an exhaustive review of all aspects of a public health approach to the use of ART, nor is it intended to function as an ART primer. However, in the absence of universally accepted treatment guidelines for HIV-2, the authors seek to provide their own recommendations, based on the available literature, HIV-2 treatment meetings, discussions with colleagues from major HIV-2 treatment centers in Europe and Africa, and from personal experiences between 2003–2010 at the Genito-Urinary Medicine clinic at the MRC Laboratories in The Gambia, where ART was provided to HIV-2 infected people.

## 2. Selecting First- and Second-Line ART Regimes in HIV-2

### 2.1. Natural Polymorphisms and Patterns of Genotypic Resistance in HIV-2

The most crucial difference between HIV-1 and HIV-2 when considering suitable ART regimes is the lack of susceptibility of the latter to what would now be called first-generation NNRTIs, nevirapine, and efavirenz [19, 20]. The natural resistance of HIV-2 to these drugs is due to differences in the amino acid residues that make contact with the NNRTI in the binding pocket of HIV-1 and HIV-2, particularly the Y181I and Y188L natural polymorphisms seen in HIV-2, which significantly reduce NNRTI binding [7]. It is worth noting that HIV-1 mutations at these positions result in complete resistance to NNRTIs [20, 21]. Although etravirine is reported to have more activity against HIV-2 than previous NNRTIs, the presence of L181 and other structural differences in the HIV-2 NNRTI-pocket makes HIV-2 naturally resistant to etravirine as well (reviewed in [22, 23]).

Nucleos(t)ide reverse transcriptase inhibitors (NRTIs) have a similar potency in both HIV-1 and HIV-2. Earlier *in vitro *work demonstrating lower potency for zidovudine (AZT) [24] appeared to be an artifact of the assay used, and more recent work demonstrates similar potency in both HIV-2 and HIV-1 [25]. The development of NRTI resistance in HIV-2 shares many parallels with that in HIV-1, although key differences are worth highlighting that are of clinical relevance. The M184V mutation occurs rapidly, both *in vitro* [26] and *in vivo* [27] in approximately 83% of patients failing a lamivudine (3TC)-containing regimen [28, 29]. As in HIV-1, it is associated with high-level phenotypic resistance to 3TC and emtricitabine (FTC) in HIV-2-infected individuals [30]. Although HIV-1 and HIV-2 share some classic NRTI resistance patterns, the preference for alternative resistance pathways has been noted, in addition to unique resistance patterns in HIV-2. AZT resistance in HIV-1 occurs via two well-documented pathways. The most common and preferred pathway is marked by the accumulation of the six thymidine-associated mutations (TAMs): M41L, D67N, K70R, L210W, T215Y, and K219Q/E [31]. The less common pathway is via the Q151M mutation, which also tends to develop later. The TAM mutations are conspicuously absent in the AZT resistance profiles of HIV-2 patients [27, 28, 32–35]. In place of TAMs, AZT resistance in HIV-2 often involves the Q151M mutation, which occurs faster and with a much higher frequency and potency than in HIV-1 [30, 36]. Considering that this mutation causes multi-NRTI resistance in HIV-2, its high frequency raises real concerns [29, 37]. Unlike HIV-1 where K65R leads to TDF, ABC, ddI, and d4T resistance [38], K65R in HIV-2 does not cause phenotypic resistance to TDF, but causes high-level resistance to 3TC and FTC, and low-level resistance to ddI [30]. Current evidence suggests that this mutation rarely occurs in HIV-2 except during suboptimal mono- or dual-therapy with NRTIs that mostly do not include TDF [27, 39, 40], where it is often associated with the Q151M mutation. There is, however, limited experience with widespread use of TDF containing regimes in first-line HIV-2 ART, and firm conclusions on a reduced frequency of K65R in HIV-2 infection cannot be drawn with confidence. Of note, in a recent Senegalese study, two (of 23) patients exhibited K65R mutations at follow-up while on an AZT/3TC/indinavir (IDV) regimen, although in one case the mutation was present at baseline prior to commencing ART [35]. These data together with the phenotypic data by Smith et al. [30] indicate that K65R arises primarily due to 3TC/FTC pressure in HIV-2. The potential fragility of currently available NRTI backbones for use in HIV-2 therapy is highlighted by the finding that Q151M combined with K65R or M184V results in high-level AZT and 3TC resistance, whereas the presence of all three mutations in combination confers class-wide NRTI resistance, although it should be noted that these mutations result only in low level resistance (4-5 fold) to d4T and TDF [30]. Lastly, the L74V mutation is rarely documented in HIV-2 [41], with one report of L74I in an HIV-2-infected patient on dual therapy (which included ddI) [40].

HIV-1 and HIV-2 proteases have an amino acid sequence similarity of about 50%, substantially less than that observed in their reverse transcriptase enzymes. These sequence differences are reflected in very distinct natural polymorphisms in the HIV-1 and HIV-2 proteases, most of which occur outside the functionally relevant areas [42]. Several HIV-2 natural polymorphisms correspond to drug resistance mutations in HIV-1. These include the major drug resistance mutation M46I, conferring resistance to indinavir (IDV), and several minor mutations, L10V, V32I, M36I, I47V, A71V, and G73A, that may decrease the activity of nelfinavir (NFV) and amprenavir (APV) [32, 43–46]. Several *in vitro* cultural and cell-free assays using individual PIs have suggested that while IDV, saquinavir (SQV), lopinavir (LPV), darunavir (DRV), and tipranavir (TPV) may exert full activity against wild-type HIV-2 [47–52], NFV and APV show a significant reduction in activity [46, 53]. A more in-depth study (kinetic inhibition assays) has shown that LPV, SQV, TPV, and DRV exhibit the highest potency in this order and that atazanavir (ATV), NFV, and APV show the lowest potency, respectively [54]. The data on TPV are however controversial, with other studies showing several fold lower potency when compared to LPV, SQV, and DRV [55, 56]. Once protease inhibitor- (PI-) based ART starts, this background of minor mutations may result in rapid acquisition of a multi-PI resistance phenotype [45, 46]. 

In HIV-1, PI resistance is associated with the accumulation of four or more resistance mutations in the protease gene, though major mutations can cause substantial resistance on their own [57]. HIV-1 and HIV-2 have similar PI resistance mutations [33, 43, 45, 46, 53], with a few mutations unique to HIV-2 [20, 45, 46]. The presence of certain natural polymorphisms in HIV-2 can reduce the time to resistance in some cases [34, 46]. For instance, I47A and V32I are associated with high-level resistance to LPV/ritonavir (LPV/r) in HIV-1 [58–60], and V47A is associated with phenotypic resistance to LPV/r in HIV-2 [61]. In HIV-1, the emergence of the LPV/r mutation I47A is a two-step process (I → V → A), whereas in HIV-2 it can occur in a single step from V → A [61]. In addition, V321 is present naturally in HIV-2. Therefore while LPV/r resistance in HIV-1 requires the acquisition of V32I and a two-step process to acquire I47A, only a one-step change in HIV-2 is required, making the development of this mutation easier and faster in HIV-2 [34].

### 2.2. Potential Options for Standardized First- and Second-Line Regimes in Resource Poor Settings

Until recently, most studies reporting antiretroviral use in HIV-2 patients were from European cohorts, and often involved mono- or dual-therapy and multiple heterogeneous regimens [28, 32, 33, 40, 62]. Due to the recent availability of ART in West Africa, data from the use of standardized first-line ART regimens in these cohorts are now appearing [34, 35, 63], although the numbers are still relatively small when compared to the HIV-1 literature. Given the lack of utility of NNRTIs in HIV-2, a key issue in choosing first-line ART regimes in HIV-2 infection is the question of whether triple NRTI regimens are a viable, safe, and efficacious option. The appeal of this approach lies in its lower pill burdens and reservation of PIs for second-line therapy, maintaining parallels with HIV-1 protocols. Prior to the development of a heat stable formulation of ritonavir, and in settings where this is not yet available, cold chain requirements also argue for a PI-sparing regimen. Unfortunately studies to date suggest that these regimes, including those with TDF, perform poorly in HIV-2 [28, 62, 64] and in our opinion should be avoided, although in certain specific circumstances they may represent the best balance of risk and benefit (see special populations, below). One case of a patient achieving viral suppression on a quadruple NRTI regimen (d4T/3TC/ABC/TDF) has been reported [62], although clearly more evidence is needed to conclude that such a regimen is superior to using triple NRTIs in HIV-2. The principal challenge of the PI-sparing nucleoside regimens in HIV-2 is the rapid development of the Q151M pan-NRTI resistance mutation [36]. Unlike the case in HIV-1 where this typically arises only after multiple other resistance mutations have developed, in HIV-2 it is one of the earliest and most common NRTI mutations (after those at the M184 locus), especially after mono-/dual-/triple-NRTI treatment [28, 32, 33, 40, 62] and compromises the entire regimen [36]. Triple nucleotide regimes containing ABC (in the absence of TDF) have also been shown to rapidly select for K65R in HIV-2 patients [64].

Our experience at the MRC Gambia [34] and that of others [62, 65] suggest that the combination regimen of AZT/3TC and LPV/r has a reasonable chance of success as a first-line regime for HIV-2 infection [34, 62, 65]. The use of an AZT/3TC backbone with unboosted IDV, however, has been shown to result in a high proportion of ART failures and accumulation of resistance mutations in a Senegalese cohort [35, 56]. ABC/3TC, TDF/FTC, and ddI-based regimens have the advantage of daily dosing and show potential for success, although in our opinion there is currently insufficient experience with those combinations in HIV-2 to draw firm conclusions. Moreover the inclusion of appropriate PIs in HIV-2 regimes will necessitate twice daily dosing in most circumstances, reducing the benefit of once-daily nucleoside analogue dosing. Didanosine also has a rather unique set of advantages and disadvantages as part of ART regimes. It should generally be taken on an empty stomach while other antiretrovirals, in particular TDF, should be taken with food, adding to regimen complexity. We believe ddI is less well tolerated than ABC, AZT, TDF, or 3TC and that this could threaten patient adherence to the overall regimen. Its use *with* TDF is relatively contraindicated because of the negative impact this combination has on CD4 cell counts and the increased risk of viral failure [66], even at the appropriate 250 mg dose [64]. Although no head-to-head comparisons have been performed in HIV-2-infected individuals, the HIV-1 literature suggests that an NRTI backbone of TDF/FTC (or 3TC) may, on the grounds of efficacy and tolerability, be a better choice than AZT/3TC [67, 68] or ABC/3TC [69, 70]. On that basis, TDF may be desirable in first-line treatment in spite of its greater cost when compared with AZT (see Table 2), although the low yet measurable risk of renal toxicity with TDF use, particularly in settings where renal monitoring may be limited, is grounds for concern [71]. Tolerability issues should also be considered. If a patient does not tolerate AZT in first-line treatment other alternatives (including d4T) could be used, following the same protocols as are used for AZT intolerance in HIV-1-infected individuals; however an equivalent substitute for TDF in the face of resistant virus is not easy to find in the event of TDF intolerance. Given that the prevalence of HLA B*5701 is low in black African individuals [72], with HLA*B5703 being the only B57 subtype found in populations in Guinea-Bissau [73], the risk of ABC hypersensitivity, if ABC/3TC is used first line without the ability to determine HLA type, may not be of great concern in sub-Saharan Africa.

As mentioned above, PI options are constrained in HIV-2 as a result of natural polymorphisms that support PI resistance. In addition, unboosted PI regimens should be avoided as they tend to perform poorly [29, 35, 40, 56]. While good clinical outcomes with LPV/r have been observed [34, 65], *in vitro* data [54] suggests that SQV/r would be a reasonable first-line PI [54] too, while IDV/r may also be effective [28]. DRV/r would appear to be reliable based on *in vitro* data, although at present there is insufficient data to justify its use as the preferred first-line PI for HIV-2 given its higher cost (see below). Boosted ATV cannot be recommended in HIV-2 [54, 55], and given the conflicting results on the use of boosted TPV [54–56], it also cannot be recommended for use until further studies confirm its efficacy. 

Second-line therapy should be considered in drafting treatment guidelines for first-line ART, as initial regimen choices narrow later treatment options. In the absence of viral load monitoring, resistance should be anticipated at the time of regimen change, and we make the assumption that resistance test results will not generally be available. Two fundamentally different strategies in ART are to increase potency up-front in order to minimize failure rates, or to hold potent antiretrovirals in reserve in order to mitigate the impact of failure of the first-line. Knowledge of typical mutations selected for during failure allows one to optimize sequential treatment, although HIV-2 is much less well studied in terms of the frequency with which various sequential regimens select for resistance mutations. 

With regards to NRTIs, extensive resistance should be assumed to include the Q151M, K65R, and M184V, depending on the NRTIs employed in the first-line ART. While ABC is probably an option where only the Q151M mutation is present, it would be compromised in the presence of K65R and M184V [30]. Older NRTIs including AZT and ddI are not likely to have much residual potency in the face of these mutations; however TDF and d4T might retain sufficient potency in this setting [30]. The argument that the M184V mutation carries a substantial fitness cost has not been demonstrated as clearly in HIV-2 as it has in HIV-1 [75], nonetheless as it occurs in the highly conserved YMDD motif within the reverse transcriptase's active site [76], we believe it is likely to affect fitness similarly, and we recommend continued exposure to 3TC or FTC in order to maintain the M184V. 

Based on the resistance data described earlier, recommended first-line boosted PIs for HIV-2 in resource limited settings are LPV/r, SQV/r, and possibly IDV/r. It appears that HIV-2 V47A mutants, selected for by failure on a LPV/r regimen, retain susceptibility to other PIs and are in fact hypersusceptible to ATV and SQV [61]. We find that this makes SQV/r an attractive choice for second-line therapy to follow up LPV/r-based ART in HIV-2.

Given the more limited range of effective antiretrovirals, both biologically and as a consequence of HIV-2's disproportionate prevalence in the resource-limited settings of West Africa, second-line treatment in HIV-2 becomes markedly challenging. Going back to the broader question of strategy, we support a boosted PI in the first regimen because we believe that failure rates on triple NRTI regimens are unacceptable. Nonetheless we recommend TDF be held in reserve to lend potency to second-line treatment.

### 2.3. HIV-1/HIV-2 Dual Infection

Co-infection with both HIV-1 and HIV-2 occurs in countries where both viruses circulate. Although progression, as implicated by higher viral loads, is driven by HIV-1 in the majority of dually infected individuals [77], this is not always the case [34]. Treatment of dually infected individuals should be carried out using an HIV-2 regimen, to ensure that the drugs used can effectively treat both viruses [34, 78]. Given that the HIV-2 plasma viral load is usually undetectable or low in dual infections, it might seem reasonable to treat and monitor only HIV-1 (discussed in [79]). In our opinion this represents a dangerous strategy, as even with an undetectable baseline HIV-2 VL, the risk exists that as HIV-1 is controlled and CD4+ T-cell targets expand, the potential for HIV-2 replication will also increase [78]. In addition, we have successfully treated eight dually infected individuals on an HIV-2 regimen of AZT/3TC/LPV/r achieving complete suppression of both viruses for more than three years [34]. 

Taken together, Tables 1a and 1b show possible combinations that would be likely to optimize control of HIV-2 in mono- and dual-infections, across two regimens, with the authors' preference given in bold.

### 2.4. Other Agents

While some newer agents developed for use against HIV-1 show no activity against HIV-2 and other products are currently unrealistic options in resource poor settings, they warrant discussion even if they lie far outside the protocols and budgets of West African treatment programs currently. With potentially increasing numbers of HIV-2 infected patients with first-line (and perhaps second-line) regimen failures in West Africa, increasing experience with the use of newer agents in salvage therapy in European settings and, hopefully, the costs of newer agents dropping over time, HIV-2 ART guidelines will require frequent reconsideration and updates.

Two types of entry inhibitors, fusion inhibitors (FI) and coreceptor binding inhibitors, have been approved for HIV therapy. Enfurvitide (T20), a fusion inhibitor currently licensed for use in HIV-1, has been found to have no activity against HIV-2 [8] which is not surprising given that HIV-1 and HIV-2 only share an amino acid sequence similarity of less than 30%–40% in the Env protein [80]. Maraviroc, a coreceptor binding inhibitor, works by blocking the CCR5 receptor, thereby inhibiting the virus from further conformational changes that will allow fusion with the host membrane. The activity of maraviroc against HIV-2 has not been formally tested, but since this drug binds to the CCR5 receptor, it should work against R5-tropic HIV-2 viruses [81, 82]. A recent case report demonstrates the inclusion of maraviroc in a regime used successfully to control resistant HIV-2 infection [83]. However, the ability of HIV-2 efficiently to utilize other coreceptors may limit the effectiveness of these antagonists in HIV-2 treatment [84]. Another potential concern is the switch or emergence of X4-tropic viruses, which is associated with faster disease progression [84]. Although R5 to X4 switch has only been reported in a few HIV-2-infected individuals [85], a limited number of X4-tropic viruses have been isolated from symptomatic patients [80].

Integrase inhibitors (INIs) work by interfering with the insertion of HIV DNA into host DNA and raltegravir (RAL), the first licensed INI for HIV-1 therapy, appears to be safe and efficacious in both ART naïve [86] and ART-experienced patients [87]. Despite the 40% heterogeneity in HIV-1 and HIV-2 integrase genes, the functionally important motifs (the catalytic triad DDE, the HHCC, and RKK) are 100% conserved in HIV-1 and HIV-2 [88, 89]. *In vitro* susceptibility of 14 clinical HIV-2 isolates, as well as HIV-2 ROD, to RAL, has showed similar activity for HIV-1 and HIV-2 [88]. *In vivo* studies on highly treatment-experienced HIV-2-infected individuals, two with group A [88, 90] and one with group B [91], showed promising results, with viral loads reduced to undetectable results, when RAL was used in combination therapy. HIV-2 resistance to RAL *in vivo* occurs via the N155H mutation [91] which is also associated with phenotypic resistance against RAL in HIV-2 [92]. However, these HIV-2 N155H mutants, like the M184V mutation in the reverse transcriptase, are much less fit than the wild type [75, 92]. This loss in replicative capacity can be exploited when viral suppression is no longer a realistic goal of therapy, and maintaining these mutations through continued selective pressure can slow disease progression.

## 3. Special Circumstances

### 3.1. Pregnancy

While the risk of HIV-2 transmission in pregnancy only reaches about 4% (including breast milk transmission) [93], clinical and *in vitro* data would suggest that AZT monotherapy as part of a prevention of mother to child transmission program poses a considerable threat to the mother, and to the child in the event of infection, of selecting for the Q151M mutation with subsequent pan-NRTI resistance [36]. Boosted PI-based ART through the latter two trimesters of pregnancy and the breastfeeding period should be the mainstay of vertical transmission prevention; however boosted PIs may result in greater nausea or insulin resistance in a small number of patients [94].

Dosing of PIs in pregnancy is not well validated, with evidence of reduced plasma concentrations with several agents, especially when used unboosted [95, 96]. Recent findings suggest that in the absence of TDM, LPV/r dose should be increased 50% in the second and third trimesters of pregnancy [97]. SQV/r is probably effective at its standard dose of 1000/100 mg twice daily [98, 99] and IDV/r at its standard dose of 800/100 mg twice daily may be adequate, but further clarification is required [100]. As with HIV-1, concerns exist about the use of TDF as part of the nucleoside backbone during pregnancy potentially interfering with bone mineralization, although it has not as yet been associated with congenital abnormalities [101].

### 3.2. Tuberculosis (TB) Coinfection

TB is endemic in West Africa, and the problematic drug interactions between PIs and rifampin are well known, with induction of the cytochrome P450 system by rifampin resulting in accelerated metabolism of PIs, making effective dosing of the PIs more difficult to achieve. Provision of rifabutin as part of TB therapy for HIV-2 co-infected patients would therefore be ideal, allowing for the more predictable pharmacokinetic interactions between LPV/r and rifabutin. However TB treatment protocols, particularly where TB and HIV treatments are managed by different health care providers, might not adopt rifabutin as a result of cost or other considerations. In this case, where ART cannot be safely deferred, a PI-sparing regimen may represent the best balance of safety and efficacy for HIV-2/TB co-infected patients. Increasing the PI dose, for example doubling the dose of LPV/r, may be an alternative, although achieving therapeutic drug levels with tolerable dosing of LPV/r appears challenging [102, 103]. If a triple or quadruple NRTI regime is used, ART should be reassessed once TB treatment is completed and the patient switched to a boosted-PI regimen.

### 3.3. Chronic Hepatitis B (HBV) Co-Infection

Chronic HBV infection is common in West Africa, with prevalence rates of 8%–20% [104–106]; as a consequence many individuals infected with HIV-2 can be expected to have chronic HBV co-infection and a substantial proportion is likely to have high HBV viremia. Unlike the epidemiology in Western countries, most HBV transmission occurs between children and is not due to shared risk factors for transmission as between sexually active or intravenous drug using adults [107]. Screening protocols for comorbidities in HIV care settings in HBV endemic countries should include HBsAg, either for all new patients or at a minimum for those with evidence of liver disease, such as transaminitis. Where chronic HBV is present, ideally TDF/FTC or TDF/3TC should be in the first-line ART regimen [108], although this recommendation may be difficult to follow where HBV diagnostics are limited. Moreover it introduces another layer of complexity into protocol-based sequential ART. Clearly these issues would be addressed if TDF/FTC (or 3TC) were adopted as the preferred NRTI backbone, although it may be necessary to maintain them in succeeding regimens, regardless of the addition of other agents, to avoid the risk of HBV “flare” arising with their discontinuation [109].

### 3.4. Childhood

Children with HIV-2 infection present many of the same challenges as those with HIV-1 infection, such as concerns about dosing, formulations, and specifically TDF toxicity [110]. The principal differences, that vertical HIV-2 transmission is distinctly less common and that it is not rare for perinatal HIV-2 infections to present in teenagers, do not argue for any specific differences in their management compared to children with HIV-1, beyond their antiretroviral regimen being appropriate for HIV-2 as described above for adults.

## 4. Operational Issues

Endemic HIV-2 brings with it complications in terms of program management in West Africa beyond the necessary alterations in the antiretroviral therapy protocols, specifically that it complicates HIV testing and management of both stocks and staff. 

### 4.1. Diagnosis

Testing to distinguish HIV-1 from HIV-2 and dual infection can be complicated and expensive due to the presence of cross-reactive antibodies and strain differences [111, 112]. Screening tests need high sensitivity for HIV-2, while confirmatory testing may require multiple steps in order to reliably distinguish between HIV-1, HIV-2, and HIV-1/HIV-2 dual infection, detailed review of which is beyond the scope of this paper. The alternative to these more demanding and elaborate testing protocols is misdiagnosis, primarily over-diagnosis of HIV-1/HIV-2 dual infection, resulting in HIV-1 monoinfected people going onto more expensive and cumbersome PI regimens. Diagnostic clarity therefore is a trade-off between higher upfront costs in testing and savings over the longer term in pharmaceuticals, although no rigorous analysis of costs has yet been made in this context. Misdiagnosis that results in HIV-2 and dually infected patients going on treatment that ignores their HIV-2 carries a greater risk, as discussed earlier.

### 4.2. When to Start

Compared with HIV-1, more patients with HIV-2 will be long-term nonprogressors or slow progressors. Although this could be used to argue for a later CD4-driven initiation of ART, it has been demonstrated that immunological recovery on ART is worse in HIV-2 compared with HIV-1 [113] and excessive delay in initiating ART may carry long-term negative immunological consequences. While the authors support initiating treatment for HIV-2 below a CD4 count of 350/mm^3^ or possibly higher, instead of below 200/mm^3^, it may be operationally awkward to apply different CD4 cut-offs for starting ART in HIV-1 and HIV-2 where CD4-driven initiation of therapy has not yet advanced to the earlier thresholds.

### 4.3. Monitoring

There is little evidence to suggest that monitoring of patients on ART should be any different for HIV-2 than for HIV-1. In practice the lack of a commercially available viral load assay [114] makes viral load measurements harder to obtain for HIV-2. CD4 recovery has been found to be blunted in HIV-2 [113]; combined with the more limited treatment options for HIV-2 this argues against considering a lack of substantial CD4 gains on ART to be a failure. Other immunologic criteria, including a drop from peak or a return to baseline CD4, may not be any worse for monitoring response to treatment in HIV-2 than in HIV-1.

### 4.4. Stock Management

In terms of stock management, the more complicated the program, the more difficult it will be to avoid stock shortages and wastages. Endemic HIV-2 complicates the program. The numbers of HIV-2 patients needing ART are harder to estimate and may vary with changes in testing algorithms (see Section 4.1). As HIV-2 patients on ART will represent a small minority of a program or project's total number of HIV patients, small fluctuations in their number result in disproportionately large fluctuations in utilization rate, a situation that is further exacerbated for second-line treatment and pediatric cohorts. Stock management for pediatric cohorts with their heterogeneity in terms of weight, physical maturity, and ability to swallow pills is particularly difficult, and pediatric HIV-2 cohorts are likely to be extremely small. Partial standardization across both HIV-1 and HIV-2 regimens, for example using the same NRTIs regardless of HIV type or using the same PI for HIV-2 first-line treatment that one uses for HIV-1 second-line treatment, may simplify stock management and reduce shortages and wastage. 

Another factor affecting ART choices in West Africa is cost. While costs can be expected to vary over time and between countries or regions, representative daily costs for several combinations, primarily from West Africa in 2010, are given in Table 2, although neither the costs nor the ratios of costs that follow should be presumed to be static. Compared with AZT, TDF and ddI are 2-3 times and ABC 4-5 times as expensive, while d4T costs half to one-third as much. The most expensive part of the regimen is the boosted PI, and this is also the main source of cost differences between regimens. Compared to coformulated LPV/r, SQV/r is 5–7 times and DRV/r 14–23 times as expensive, while IDV/r is approximately of the same cost. Comparisons of costs should also take efficacy into account. Depending on the model and assumptions this may result in medicines with a higher daily cost being more cost-effective, as has been recently shown for TDF in first-line ART in India [115].

### 4.5. Training and Protocol Development

The differences in recommendations between HIV-1 and HIV-2 and the dosing complications, particularly with TB cotreatment and in late pregnancy, pose further challenges to front line staff involved in program implementation in the HIV-2 endemic areas of West Africa. More complicated protocols call for more detailed training of staff. Greater diagnostic ambiguity and a broader range of ART regimens require more complete medical records. Finally patients getting information from various sources, especially long-term nonprogressors, need additional counseling to understand their disease.

## 5. Summary Recommendations

West African and other programs faced with HIV-2 patients need locally adapted protocols for testing, treatment, monitoring, and stock management in order to be effective. With regards to treatment, the delivery of optimal therapy should be a program goal, and although more complicated, it is achievable within a public health framework, with nurse-led clinics, even where infrastructural or staffing deficits may exist. For adults with HIV-2 or HIV-1/HIV-2 dual infection without access to ART susceptibility testing, optimal antiretroviral therapies for first- and second-line treatment are suggested in Tables 1a and 1b. It is hoped that these recommendations will rapidly become obsolete as other agents and drug classes come into wider use in West Africa, and prospective randomized controlled trials of ART in HIV-2 provide more reliable indications of the suitability of specific regimens.

## Figures and Tables

**Table:** (a) Potential first- and second-line NRTI backbones for HIV-2 and dual infection

First-line	Second-line
**AZT/3TC**	**TDF/AZT/3TC or FTC**
TDF/FTC or 3TC*	TDF/AZT/3TC or FTC
ABC/3TC	TDF/AZT/3TC or FTC

*FTC and 3TC are assumed to be essentially equivalent in the table, despite FTC's possible superiority and 3TC's possibly lower cost.

**Table:** (b) Potential first- and second-line PIs for HIV-2 and dual infection

First-line	Second-line
**LPV/r**	**SQV/r** or DRV/r
SQV/r	LPV/r or DRV/r
IDV/r	LPV/r or SQV/r or DRV/r

**Table 2:** Representative daily costs of selected antiretrovirals in West Africa in 2010 [74]. All values represent amounts paid since 01/01/2010 in West Africa except where otherwise noted, in which case the nearest equivalent in terms of year of purchase and income was used.

Drug	Cost per day^a^
AZT/3TC	$0.28–$0.36
d4T/3TC	$0.12
ABC/3TC	$1.38^b^
ddI (400 mg buffered)	$0.79
3TC	$0.08–$0.10
TDF	$0.72^c^
TDF/FTC	$0.87–$0.88^c^
LPV/r	$1.24–$1.56^d^
IDV	$0.96^d^
SQV	$7.20^e^
DRV	$22.12–$28.40^f^
Ritonavir (100 mg bd)	$0.22–$0.96^d^

^a^Costs are given in US dollars for standard doses given twice daily or daily in the case of TDF, ddI, ABC/3TC, and TDF/FTC

^b^Dominican Republic; 2008

^c^Republic of South Africa, Somalia

^d^2009

^e^Egypt

^f^Bulgaria, Jamaica; 2009.
